# Differential effects of innate immune variants of surfactant protein-A1 (SFTPA1) and SP-A2 (SFTPA2) in airway function after *Klebsiella pneumoniae* infection and sex differences

**DOI:** 10.1186/s12931-018-0723-1

**Published:** 2018-02-03

**Authors:** Nithyananda Thorenoor, Xuesheng Zhang, Todd M. Umstead, E. Scott Halstead, David S. Phelps, Joanna Floros

**Affiliations:** 1 Center for Host defense, Inflammation, and Lung Disease (CHILD) Research, Department of Pediatrics, The Pennsylvania State University College of Medicine, Hershey, PA 17033 USA; 2 Pulmonary Immunology and Physiology (PIP) laboratory, Department of Pediatrics, The Pennsylvania State University College of Medicine, Hershey, PA 17033 USA; 3 Department of Obstetrics and Gynecology, The Pennsylvania State University College of Medicine, Hershey, PA 17033 USA; 4 Departments of Pediatrics and Obstetrics and Gynecology, Evan Pugh University Professor in Cellular and Molecular Physiology, 500 University Drive, P.O. Box 850, Hershey, PA 17033-0850 USA

**Keywords:** Surfactant protein A1 and A2, Forced oscillation technique (FOT), Infection, Airway hyperreactivity

## Abstract

**Background:**

Surfactant Protein-A (SP-A) is a major protein component of surfactant and plays a role in surfactant-related functions and innate immunity. Human SP-A consists of two functional genes, SFTPA1 and SFTPA2, encoding SP-A1 and SP-A2 proteins, respectively and each is identified with numerous genetic variants. These differentially enhance bacterial phagocytosis, with SP-A2 variants being more effective than SP-A1.

**Methods:**

Lung functions of humanized transgenic (hTG) mice that carry different SP-A1 and SP-A2 variants or both variants SP-A1/SP-A2 (6A^2^/1A^0^, co-ex), as well as SP-A knockout (KO), were studied. The animals were connected to a flexiVent system to obtain forced oscillation technique (FOT) measurements and the data were analyzed using various models. Lung function was assessed after infection (baseline) and following inhaled methacholine concentrations (0–50 mg/mL).

**Results:**

Here, we investigated the role of SP-A variants on airway function after *Klebsiella pneumoniae (Kp)* infection (baseline) and following inhaled methacholine. We found that: 1) in the absence of methacholine no significant differences were observed between SP-A1 and SP-A2 variants and/or SP-A knockout (KO) except for sex differences in most of the parameters studied. 2) In response to methacholine, i) sex differences were observed that were reverse of those observed in the absence of methacholine; ii) SP-A2 (1A^3^) gene variant in males exhibited increased total and central airway resistance (Rrs and Rn) versus all other variants; iii) In females, SP-A2 (1A^3^) and SP-A1 (6A^2^) variants had similar increases in total and central airway resistance (Rrs and Rn) versus all other variants; iv) Allele-specific differences were observed, a) with SP-A2 (1A^3^) exhibiting significantly higher lung functions versus SP-A2 (1A^0^) in both sexes, except for Crs, and b) SP-A1 (6A^2^, 6A^4^) had more diverse changes in lung function in both sexes.

**Conclusion:**

We conclude that, in response to infection and methacholine, SP-A variants differentially affect lung function and exhibit sex-specific differences consistent with previously reported findings of functional differences of SP-A variants. Thus, the observed changes in respiratory function mechanics provide insight into the role and importance of genetic variation of innate immune molecules, such as SP-A, on mechanical consequences of lung function after infection and inhaled substances.

## Background

The initial defense against inhaled pathogens, allergens, air pollutants, and other harmful substances in the environment is performed by cells and molecules present in the lung. For example, the alveolar macrophage (AM) is the principal effector cell for innate immunity against inhaled substances and thus protects the lung from these potential hazards. The distal airways and alveoli consist of epithelial type I and type II cells. The type II cells are the source of pulmonary surfactant, a lipoprotein complex, which lines the entire surface of the alveoli, and its components play a key role in innate immunity. Surfactant protein A (SP-A) is the major protein component of pulmonary surfactant and regulates host lung defense [1].

SP-A is a major surfactant host defense component and belongs to the group of mammalian lectins and collectins which are involved in innate immunity [2, 3]. SP-A is involved in multiple AM-mediated host defense functions such as the stimulation of chemotaxis of macrophages [4], enhancement of phagocytosis of bacteria by macrophages [5–8], proliferation of immune cells [9, 10], and linkage of innate and adaptive immunity [11]. We and others have shown that in the absence of SP-A the susceptibility to pneumonia and other types of lung injury is increased [12–16]. Human SP-A consists of two functional genes, SFTPA1 and SFTPA2,encoding SP-A1 and SP-A2 proteins, respectively, and each has been identified with a number of variants [17, 18]. SP-A gene variants are classified based on their nucleotide differences within the coding sequences [17–19].

The SP-A1 and SP-A2 variants have been identified with both qualitative (i.e., functional, biochemical and/or structural) differences and quantitative (regulatory) differences. The qualitative differences include differences in their ability to stimulate phagocytosis [7, 8], bind carbohydrates [20], inhibit surfactant secretion [21], and stimulate production of TNF-α by macrophage-like THP-1 cells [22–24], as well as differences in their aggregation and oligomerization properties [21, 25, 26]. Quantitative differences include differences between the SP-A1 and SP-A2 and/or variants in basal mRNA levels and in response to dexamethasone [27–30], and differences in SP-A protein levels in bronchoalveolar lavage (BAL) fluids from different individuals [31]. It has been observed that these variants differ in their ability to affect the biophysical function of surfactant, with SP-A1 exhibiting a higher efficiency in pulmonary surfactant reorganization and thus playing an important role in lung function [32]. Differences in the proteomic expression profile of AM and the AM actin cytoskeleton have been observed between SP-A1 (6A^2^) and SP-A2 (1A^0^) variants [33, 34].

Previously, we have shown that SP-A1 and SP-A2 variants differentially enhance bacterial association with the AM, with SP-A2 being more effective than SP-A1 [7, 8]. However, this activity is differentially compromised in response to ozone-induced oxidation [35]. Together these indicate that functional differences exist between SP-A1 and SP-A2 in-vivo and that the lung microenvironment differentially affects the function of SP-A variants. Furthermore, *Klebsiella pneumoniae* infection resulted in sex-dependent survival, with females exhibiting higher survival compared to males, and the reverse after oxidative stress [36], with females exhibiting lower survival compared to males. Sex hormones were implicated in the differential survival [37].

SP-A is known to play a role in lung surfactant homeostasis, host defense, and airway function in response to bacterial infection [1, 38–41]. Mice lacking SP-A exhibit significantly increased airway hyperresponsiveness to bacterial infection compared to wild type [39], indicating that SP-A may contribute to airway/lung mechanics. Thus, we speculated that SP-A influences lung mechanics, by various mechanisms where SP-A has been shown to play a role. These may include i) regulation of gene expression in lung fibroblast [42], which could influence the lung interstitium and its properties, ii) ability to regulate alveolar macrophage gene expression [22, 24], which could alter the level of inflammation in the lung and this in turn may contribute to a change in its mechanical characteristics, and iii) effect on surfactant structure and/or function that could also have an impact on lung mechanics [25, 32, 43, 44]. Based on the available literature and specifically the differential impact of SP-A variants on surfactant characteristics [32], inflammatory processes [22, 24], and infection [7, 8, 16, 36, 45], we hypothesized that the SP-A genetic variants differentially affect lung mechanics.

In the present study, we investigated the role of two SP-A1 and two SP-A2 variants that are frequently observed in the general population [17] on lung function mechanics after *K.pneumoniae* infection in the presence or absence of methacholine challenge. For this purpose, we utilized the forced oscillation technique (FOT) to measure mechanical properties of lungs. FOT is a powerful, integrative and translational approach to study the physiological changes in the lung and provides measurements of respiratory system mechanics through the analysis of pressure and volume signals. This tool is also used to assess airway responsiveness to inhaled substances (methacholine) and other pathological conditions [46]. The data obtained from FOT measurements were analyzed with flexiware software (SCIREQ) using the single-compartment model, the constant phase model, as well as the Salazar-Knowles equation.

The findings indicated that the SP-A1 and SP-A2 variants play a crucial role in the differential outcome of airway function in males and females. SP-A variants exhibited significant sex-specific and gene-specific differences in airway function mechanics in response to infection and methacholine challenge. From our studies, we speculate that the genetic makeup of innate immune molecules such as SP-A1 and SP-A2 can differentially affect the mechanics of lung function in males and females under different conditions.

## Methods

### Animals

All mice used in the present study were 12 weeks of age. We used humanized transgenic (hTG) that carried SP-A1 (6A^2^, 6A^4^), SP-A2 (1A^0^, 1A^3^), or both variants SP-A1/SP-A2 (6A^2^/1A^0^, co-ex), as well as SP-A knockout (KO). hTG mice were generated on the C57BL6/J SP-A (KO) background [47]. The animals were raised and maintained under approved housing conditions in a pathogen-free environment, at the Penn State College of Medicine animal facility. Both males and synchronized females (with regard to the estrous cycle) were used in this study. For synchronization, dirty bedding from male cages was placed in group-housed female cages 7 days prior to infection to stimulate estrus in females. The Penn State Hershey Medical Center Institutional Animal Care and Use Committee (IACUC) approved all procedures involving animals.

### Preparation of bacteria

*K. pneumoniae* bacteria (ATCC 43816) were obtained from American Tissue Culture Collection (Rockville, MD) and prepared as described previously [16]. In brief, bacteria were grown at 37 °C for 18 h in Tryptic soy broth (TSB) media to reach stationary phase. The overnight bacterial culture was diluted until the OD_660_ was equal to 0.4, and 200 μl were used to inoculate a sub-culture in 50 ml of fresh TSB for 3 h to reach the mid-log phase of growth. The growth was stopped by keeping the subculture on the ice and serially diluted in PBS to obtain ~ 9 × 10^3^ CFU/ml. Fifty μl of a bacterial suspension containing ~ 450 CFU was used to infect mice. CFU per ml values were estimated based on the standard curve obtained at OD_660_ of the bacterial suspension.

### Infection of mice with *K. pneumoniae*

Infection was performed as described previously [16]. Briefly, humanized transgenic (hTG) mice, SP-A1 (6A^2^, 6A^4^), SP-A2 (1A^0^, 1A^3^), SP-A1/SP-A2 (6A^2^/1A^0^, co-ex) and SP-A (KO) male and female mice (*n* = 4 for per group) were anesthetized with a mixture of ketamine and xylazine and infected with *K. pneumoniae* (~ 450 CFU/mouse) in 50 μl of PBS intratracheally for 18 h.

### Respiratory mechanics

Parameters of lung function were measured using the forced oscillation technique (FOT) and a computer-controlled flexiVent FX ventilator (SCIREQ, Montreal, Canada) as previously described by McGovern [46]. Briefly, after 18 h of infection, the mice were anesthetized with a mixture of ketamine and xylazine. The trachea was cannulated and mice were connected to the flexiVent via cannula. Oxygen-containing 2% Isoflurane was used to ventilate the lungs at a rate of 150 breaths per minute (BPM) with a positive end-expiratory pressure (PEEP) of 3cmH2O. To block spontaneous respiratory movement, a non-polarizing paralytic (vecuronium bromide) was used. Manual pressure-volume loop (PV) and deep inflation scans were performed to obtain baseline parameters, followed by methacholine response. Methacholine (acetyl-β-methylcholine chloride, Sigma-Aldrich, St Louis, MO) doses were prepared prior to the start of the experiment and administered using the flexiVent Aeroneb fine particle nebulizer in DPBS for 10 s per dose with concentration ranging from 0 to 50 (0, 1.56, 3.13, 6.25, 12.5, 25 and 50) mg/mL. The script used for the inhaled dose response included two Deep Inflation scans followed by 12 repeats of alternating SnapShot (sinusoidal – single frequency forced oscillation waveform) and Primewave (broadband – multi-frequency forced oscillation waveform) scans were performed for baseline (18 h after infection) measurement and for each dose methacholine challenge.

Data were analyzed with flexiware software (SCIREQ) using the single-compartment model, the constant phase model, and the Salazar-Knowles equation. The single-compartment model was used to evaluate total respiratory resistance (Rrs) and elastance (Ers). The constant phase model allows measuring the partitioning of the responses within the lungs [48]. The constant phase model fits into the equation: *Z*_rs_(*f*) = R_N_ + *j* x 2π*f* x I_aw_ + (G - *j* x H)/(2π*f*)α, where Z is input impedance and expresses the combined effects of resistance, compliance, and inertance as a function of frequency (*f*); R_N_ is Newtonian “airway” resistance: I_aw_ is airway inertance and is dominated by the mass of gas in the central airways, and impedance of tissue is accounted for by G (tissue damping) and H (tissue elastance). G is closely related to peripheral airway and tissue resistance and reflects energy dissipation in the lung tissues, *j* is an imaginary number, H is tissue elastance and reflects energy storage in the tissues, α is 2/π tan^− 1^ (H/G), and *f* is respiratory frequency [48]. The PV loop was fit into the Salazar-Knowles equation to obtain static compliance (C_st_), an estimate of the inspiratory capacity (*A*) and the shape constant *k* [49]. Data were then exported to Excel for further analysis.

### Statistical analysis

The analysis of respiratory mechanics parameters and expiratory flow obtained under baseline condition (infection) and methacholine challenge was performed by one-way and two-way analysis of variance (ANOVA) followed by Bonferroni multiple comparisons correction for each experimental group with *p* value < 0.05 considered to be significant (GraphPad Prism version5; GraphPad Software, San Diego, USA). Data are expressed as mean ± standard deviation (SD).

## Results

hTG mice, SP-A1 (6A^2^, 6A^4^), SP-A2 (1A^0^, 1A^3^), SP-A1/SP-A2 (6A^2^/1A^0^, co-ex), and SP-A knockout (KO) male and female mice were infected with *K.pneumoniae*. After 18 h of infection airway lung function measurements were performed in the absence or presence of methacholine challenge as described in methods.

### Respiratory mechanics after infection (baseline)

#### Sex differences between SP-A1, SP-A2, KO, and SP-A1/SP-A2 (6A^2^/1A^0^, co-ex)

**Fig. 1:**
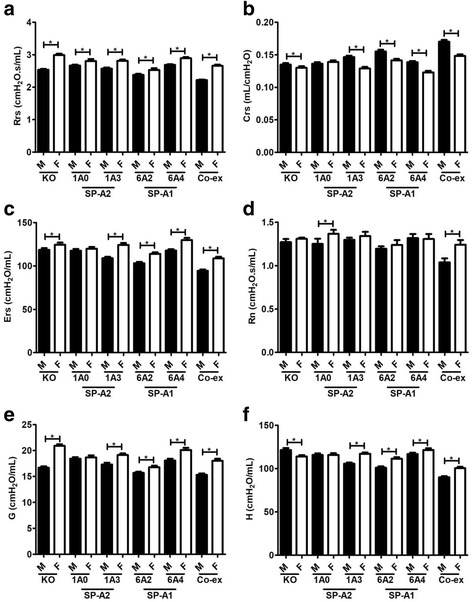
Effect of sex on airway function after *Klebsiella pneumoniae (Kp)* infection (baseline). Airway functions such as respiratory resistance (Rrs) panel **a**, compliance (Crs) panel **b**, elastance (Ers) panel **c**, and constant phase model parameters airway resistance (Rn), tissue damping (**g**), and tissue elastance (**h**), (panels **d**, **e**, **f**) were measured in SP-A1 (6A^2^, 6A^4^), SP-A2 (1A^0^, 1A^3^), SP-A1/SP-A2 (6A2/1A^0^, co-ex), and SP-A (KO) male and female mice after 18 h of infection. Data are expressed as mean ± SD (4 replicates per group). *p < 0.05

The females of all mouse lines studied showed a significant increase in total resistance (Rrs) compared to males (Fig. 1a). In contrast, all females showed a decrease in compliance (Crs) compared to all males except for the SP-A2 (1A^0^), where no significant differences between males and females were observed (Fig. 1b). For elastance (Ers) and tissue damping (G), female values were significantly greater than males for all except the SP-A2 (1A^0^) mice (Fig. 1c, e). In terms of tissue elastance (H), all females showed a significant increase compared to males except for the SP-A2 (1A^0^) that showed no sex difference and KO females showed a significant decrease compared to males (Fig. 1f). With respect to Newtonian airway resistance (Rn), the SP-A2 (1A^0^) and co-ex females showed an increase compared to males but none of the other mouse lines exhibited any significant difference (Fig. 1d).

#### Differences among mice that lack SP-A (KO) or mice that carry a different SP-A variant (SP-A1 or SP-A2)

**Fig. 2:**
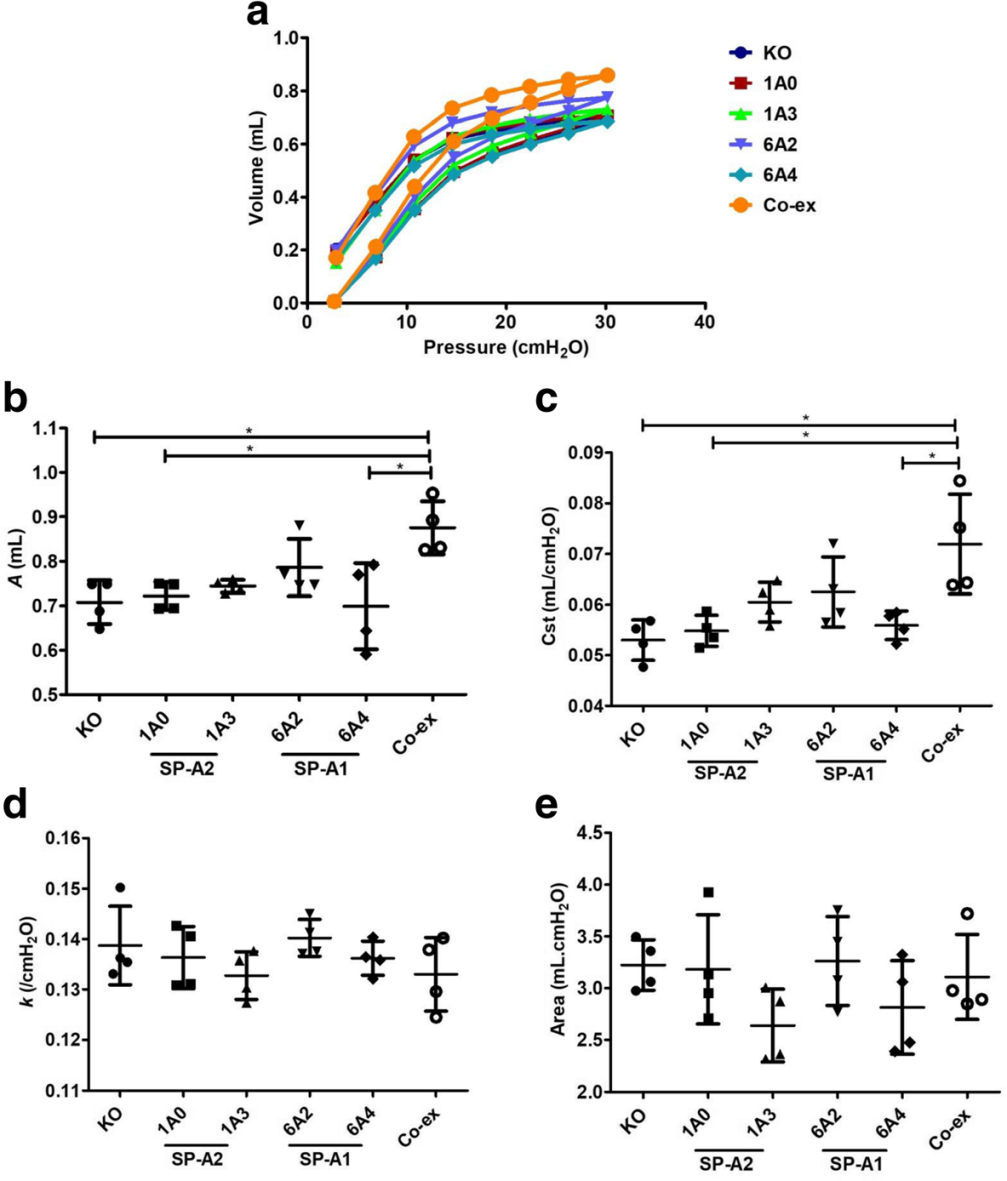
Baseline elastic properties of the respiratory system in infected males. Pressure-volume (PV) loops for male SP-A1 (6A^2^, 6A^4^), SP-A2 (1A^0^, 1A^3^), SP-A1/SP-A2 (6A^2^/1A^0^, co-ex), and SP-A (KO) mice (**a**). Parameter *A* from the exponential function used to fit the deflation limb of each PV loop [49], which provides an estimate of the inspiratory capacity shown in (**b**), static compliance (Cst) shown in (**c**), the shape constant *k* shown in (**d**), and the area between the PV loop is shown in (**e**). Data are expressed as means ± SD (4 replicates per group). *p < 0.05

**Fig. 3:**
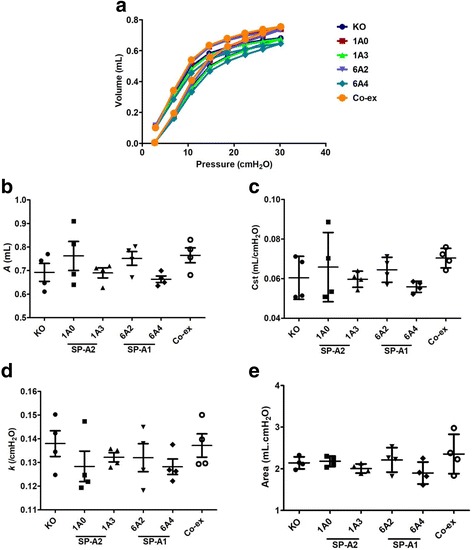
Baseline elastic properties of the respiratory system in infected females. Pressure-volume (PV) loops for female SP-A1 (6A^2^, 6A^4^), SP-A2 (1A^0^, 1A^3^), SP-A1/SP-A2 (6A^2^/1A^0^, co-ex), and SP-A (KO) mice (**a**). Parameter *A* from the exponential function used to fit the deflation limb of each PV loop [49], which provides an estimate of the inspiratory capacity shown in (**b**), static compliance (Cst) shown in (**c**), the shape constant *k* shown in (**d**), and the area between the PV loop is shown in (**e**). Data are expressed as means ± SD (4 replicates per group). *p < 0.05

Differences in respiratory properties among the mouse lines at the time of assessment (i.e. 18 h after infection) are as follows. No significant differences were observed in pressure-volume (PV) curves among the variants in either males (Fig. 2) or females (Fig. 3) after infection. Although SP-A1 (6A^2^), and co-ex males and females (Figs. 2a and 3a), and SP-A2 (1A^0^) females (Fig. 3a) exhibited a non-significant upward shift in PV curves, this did not result in any significant difference in any of the functions studied compared to other variants or KO (Figs. 2b, c & 3b, c).

#### Mice that carry a single gene variant SP-A1, SP-A2, or mice that lack SP-A versus mice that carry both SP-A1/SP-A2 variants, i.e. co-ex

Mice carrying SP-A1/SP-A2 variants (co-ex) in males, exhibited similar inspiratory capacity (parameter *A*) and static compliance (Cst) with SP-A2 (1A^3^) and SP-A1 (6A^2^), whereas the other variants exhibited a significant decrease in inspiratory capacity and static compliance compared to co-ex (Fig. 2b, c). Furthermore, the male co-ex showed significant increases in inspiratory capacity and compliance compared to KO males (Fig. 2b, c), but no other differences were found with KO in the parameters studied. In contrast, in females, there was no significant difference in inspiratory capacity and static compliance between mice carrying both SP-A1/SP-A2 gene variants, mice that carry a single gene variant, and KO mice (Fig. 3b, c).

Moreover, in the dimensionless and volume-independent shape constant *k* that describes the curvature of the curves (Figs. 2d & 3d) and in the area between the inflation and deflation limb of the PV curves (Figs. 2e & 3e), no change was observed in either males or females. This indicates no change in the intrinsic elastic properties of the respiratory system after infection among the males and females experimental groups.

In general, the data in section I indicate that in response to infection there are sex differences in most (if not all) parameters studied, with females exhibiting higher values (except in Crs).

### Respiratory mechanics after infection and in response to methacholine

#### Sex differences between SP-A1, SP-A2, KO, and SP-A1/SP-A2 (6A^2^/1A^0^, co-ex)

**Fig. 4:**
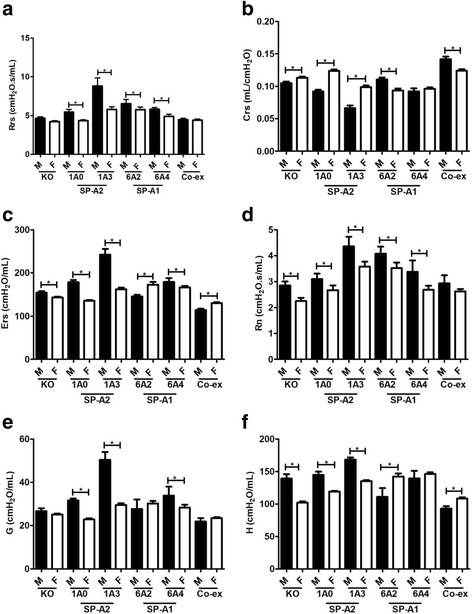
Effect of sex on airway function after infection and methacholine (50 mg/mL). Airway functions such as respiratory resistance (Rrs) panel **a**, compliance (Crs) panel **b**, elastance (Ers) panel **c**, and constant phase model parameters airway resistance (Rn), tissue damping (**g**), and tissue elastance (**h**), (panels **d**, **e**, **f**) were measured in SP-A1 (6A^2^, 6A^4^), SP-A2 (1A^0^, 1A^3^), SP-A1/SP-A2 (6A2/1A^0^, co-ex), and SP-A (KO) male and female mice after challenge with methacholine (50 mg/mL). Data are expressed as mean ± SD (4 replicates per group). *p < 0.05

Challenge with methacholine (50 mg/mL) resulted in significant sex differences in the parameters studied, i.e., respiratory resistance (Rrs), elastance (Ers), Newtonian airway resistance (Rn), tissue damping (G), tissue elastance (H), and compliance (Crs). In the **SP-A2 (1A**^**0**^**, 1a**^**3**^**) groups**, all males compared to females showed a significant increase in Rrs, Ers, Rn, G, and H respiratory functions (Fig. 4a, c, d, e, f) and a significant decrease in Crs (Fig. 4b). However, in the case of **SP-A1 (6A**^**2**^**, 6A**^**4**^**) groups**, a more diverse response was observed. Both 6A^2^ and 6A^4^ males showed an increase in Rrs and Rn (Fig. 4a, d). The 6A^2^ males showed an increase in Crs (Fig. 4b), and a decrease in H (Fig. 4f), and the 6A^4^ showed no sex difference in either parameter. The 6A^2^ and 6A^4^ males showed a decrease and increase in Ers, respectively (Fig. 4c). The 6A^4^ males also showed an increase in tissue damping (G) but no change was observed in 6A^2^ males (Fig. 4e). In **KO**, sex differences were observed with males exhibiting higher Ers, Rn, and H (Fig. 4c, d, f) and lower (Crs) values (Fig. 4b). In mice carrying both **SP-A1/SP-A2 variants (co-ex)**, males had higher Crs (Fig. 4b) and lower Ers, and H (Fig. 4c, f) values, with no sex changes observed in Rrs, Rn, and G (Fig. 4a, d, e).

#### Gene-specific variant differences

**Fig. 5:**
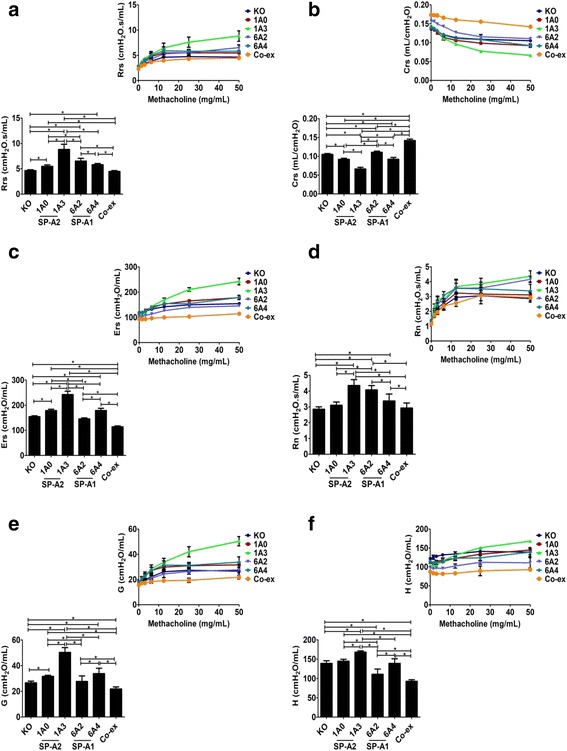
Effect of SP-A variants on airway functions after *Kp* infection and in response to methacholine challenge (50 mg/mL) in males. The Rrs, Crs, Ers, Rn, G, and H (panel **a**, **b**, **c**, **d**, **e**, and **f**), were measured in male SP-A1 (6A^2^, 6A^4^), SP-A2 (1A^0^, 1A^3^), SP-A1/SP-A2 (6A2/1A^0^, co-ex), and SP-A (KO) mice after a 50 mg/mL of methacholine challenge (bar graph). Inset: depicts the effect of dose response with increasing concentration of methacholine. Data are expressed as mean ± SD (4 replicates per group). *p < 0.05

**Fig. 6:**
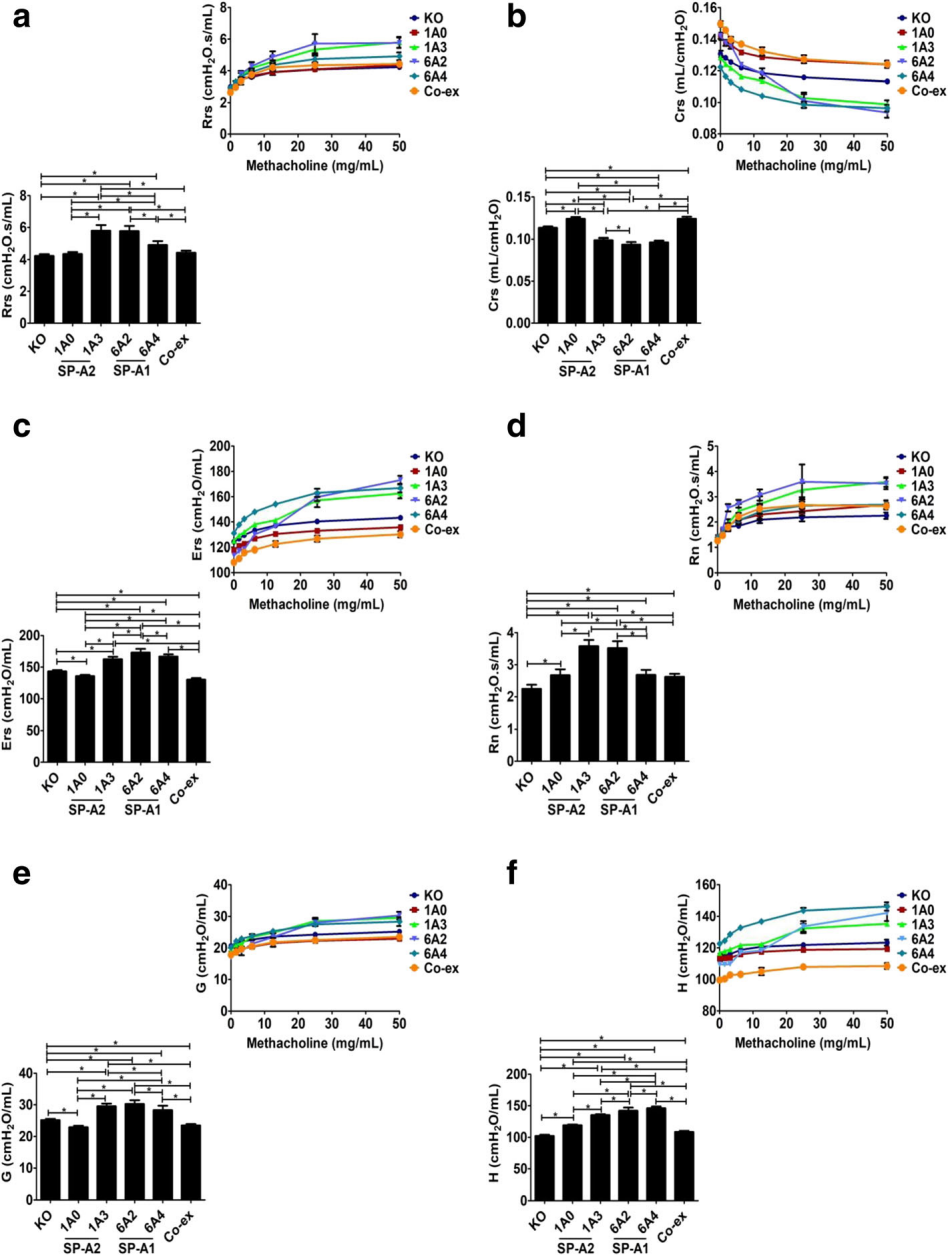
Effect of SP-A variants on airway functions after *Kp* infection and in response to methacholine challenge (50 mg/mL) in females. In female SP-A1 (6A^2^, 6A^4^), SP-A2 (1A^0^, 1A^3^), SP-A1/SP-A2 (6A2/1A^0^, co-ex), and SP-A (KO) mice, Rrs, Crs, Ers, Rn, G, and H (panel **a**, **b**, **c**, **d**, **e** and **f**)were measured after a 50 mg/mL of methacholine challenge (bar graph). Inset: depicts the effect of dose response with increasing concentrations of methacholine. Data are expressed as mean ± SD (4 replicates per group). *p < 0.05

**SP-A2:** Challenge with methacholine (50 mg/mL) resulted in SP-A2 gene-specific variant differences between 1A^0^ and 1A^3^. In both male and female mice, there was a decrease in Rrs, Ers, Rn, G, and H functions and an increase in Crs function in 1A^0^ compared to 1A^3^ (Figs. 5a-f & 6a-f).

**SP-A1:** The methacholine (50 mg/mL) challenge resulted in more diverse changes between the SP-A1 gene-specific variants in males and females. For example, the 6A^4^ mice versus the 6A^2^ mice showed a significant decrease in both males and females in Rrs and Rn (Figs. 5a, d & 6a, d), and although the 6A^4^ had a significant decrease in Crs in males, no significant changes were observed in females (Figs. 5b & 6b). In some of the other functions such as, Ers and G, the 6A^4^ males compared to 6A^2^ males showed a significant increase but the 6A^4^females compared to 6A^2^ females had the opposite result showing a significant decrease in these parameters (Figs. 5c, e & 6c, e); both 6A^4^ males and females showed a significant increase in H compared to 6A^2^ male and females respectively (Figs. 5f & 6f). Thus, although significant differences between variants of SP-A1 or SP-A2 were observed, the SP-A2 variants did not show any sex differences in the functions studied, whereas SP-A1 variants did show sex differences.

#### Differences between SP-A1 and SP-A2 variants

The **SP-A2 (1A**^**0**^**)** males and females exhibited significant functional changes compared to SP-A1 (6A^2^) variants in response to methacholine (50 mg/mL). The 1A^0^ showed a significant decrease (vs 6A^2^) for both males and females in Rrs and Rn (Figs. 5a & 6d), and a significant decrease in Crs in males, but a significant increase in females (Figs. 5b & 6b). In some of the functions such as Ers, G, and H, the 1A^0^ males showed a significant increase relative to 6A^2^ males but the females had the opposite result showing a significant decrease in these parameters compared to 6A^2^ (Figs. 5c, e, f & 6c, e, f).

However, in males no significant differences were observed between 1A^0^ and 6A^4^ in any of the parameters studied (Fig. 5a-f), but in 1A^0^ females a significant decrease was observed in Rrs, Ers, G, and H (Fig. 6a, c, e, f) and a significant increase in Crs function (Fig. 6b) compared to 6A^4^. There was no significant change in Rn values between 1A^0^ and 6A^4^ females (Fig. 6d).

The **SP-A2 (1A**^**3**^**)** males and females exhibited significant functional changes compared to SP-A1 (6A^2^) variants in response to methacholine (50 mg/mL). In males, the 1A^3^ showed a significant increase in Rrs, Ers, G, and H (Fig. 5a, c, e, f) compared to 6A^2^. However, the 1A^3^ females had opposite results in Ers and H function showing a significant decrease (Fig. 6c, f) and with no significant changes in Rrs and G compared to 6A^2^ females (Fig. 6a, e). Moreover, in Crs the 1A^3^ male had a significant decrease, and the 1A^3^ females showed a significant increase (Figs. 5b & 6b). There was no significant change in Rn values between 1A^3^ and 6A^2^ in either males or females (Figs. 5d & 6d).

The **SP-A2 (1A**^**3**^**)** also had significant changes in response to methacholine (50 mg/mL) compared to SP-A1 (6A^4^) variants for both males and females. For example, the 1A^3^ when compared to 6A^4^ showed a significant increase in both males and females in Rrs, Rn, and G (Figs. 5a, d, e & 6a, d, e), and a significant increase and decrease for H in males and females, respectively (Figs. 5f & 6f). In some other functions such as Crs and Ers, the 1A^3^ males showed a significant decrease and increase, respectively (Fig. 5b, c), with no significant changes being observed in 1A^3^ females compared to 6A^4^ (Fig. 6b, c).

#### Differences with SP-A1/SP-A2 (co-ex) or KO and SP-A1 or SP-A2 variants

i. The SP-A1/SP-A2 (co-ex) exhibited significant differences compared to different SP-A variants in several parameters in both males and females, as shown in Figs. 5 and 6. Of interest, the co-ex was similar to 1A^0^ males (Rn, Fig. 5d) and 1A^0^ females (Rrs, Crs, Rn, and G, Fig. 6a, b, d, e), as well as 6A^4^ females (Rn, Fig. 6d).
ii. The KO exhibited significant differences compared to SP-A variants as did the co-ex in several parameters for both males and females, as shown in Figs. 5 and 6. Of interest, the KO was similar to 1A^0^ males (Rn, and H, Fig. 5d, f) and 1A^0^ females (Rrs, Fig. 6a), the 6A^2^ males (Ers, and G, Fig. 5c, e), the 6A^4^ males (H, Fig. 5f), and the co-ex males (Rrs, and Rn, Fig. 5a, d), as well as the co-ex females (Rrs, Fig. 6a).

The data in section II in response to infection and methacholine challenge indicate that a) the observed sex differences are reversed from those observed in response to infection alone, and b) SP-A variants exhibit a differential impact on lung function parameters. Together these provide insight on the varied contribution of SP-A variants to lung function.

## Discussion

Surfactant protein A (SP-A) plays an important role in lung innate immunity. The SP-A variants differ in their ability to enhance association of bacteria with the alveolar macrophage (AM), and SP-A2 variants are more effective than SP-A1 [3, 7, 8]. The lung microenvironment also affects the functional activity of a given variant. For example, an increase in reactive oxygen species (ROS) differentially oxidizes the SP-A variants which consequently has an effect on their function. Oxidative stress is shown to affect SP-A2 gene-specific variants activity more than that of the SP-A1 variants [35]. In the current study, we compared the effect of SP-A1 (6A^2^, 6A^4^) and SP-A2 (1A^0^, 1A^3^) gene-specific variants on respiratory functions after *Klebsiella pneumoniae* infection and airway responses to methacholine challenge. The goal was to gain insight into the differences resulting from the presence or absence of SP-A1 and SP-A2 variants on lung function as well as elucidating the role of these variants in airway hyperreactivity. We used the forced oscillation technique (FOT) to measure mechanical properties of lungs, and measured total respiratory resistance (Rrs), elastance (Ers), Newtonian “airway” resistance (Rn), tissue damping (G- energy dissipation in the alveoli), and tissue elastance (H- energy conservation in the alveoli).

### Sex differences in response to infection (baseline) and in response to methacholine

Sex is one of the factors that differentially modulates lung function. Previously, we have shown that sex differences exist in the clinical course of mice with pneumonia combined with an oxidative stress [16, 36, 45]. Sex hormones have been shown to influence lung function, the course of the disease, and the response to environmental agents in animal models [37, 50]. In the present study, all SP-A variants exhibited sex differences in lung functions in response to infection at baseline and in response to methacholine in all of the parameters studied. The infection itself affected the respiratory mechanics, with females exhibiting a significant increase in most FOT parameters compared to males. Of interest, this pattern was reversed after methacholine challenge with males showing significant increases in nearly all FOT parameters. The increase in Rrs indicates that infection may cause an increase in resistive properties in females, but after methacholine challenge, females exhibit the opposite result. Rrs is commonly interpreted as respiratory system resistance (surrogate of airway resistance). Our data indicated that the respiratory system resistance was altered in our experimental condition. Thus, Rrs in addition to the respiratory system resistance is also coupled to the resistive properties of lung tissue (Figs. 1a, & 4a), which confirms previous findings [51]. Rn, provides a good measure of the overall resistance of the conducting airways that are dominant in proximal airways [52]. Rn was not altered after infection, except for in 1A^0^ and co-ex mice, indicating that loss of lung function after infection is likely restricted to the periphery, and may be due to small airway closure or alveolar collapse. However, Rn was altered following methacholine challenge, with males exhibiting a significant increase compared to females in all mouse lines studied except for co-ex (Figs. 1d, & 4d). This indicates that the airway is narrowing as methacholine aerosol reaches the airway smooth muscle and causing it to contract. It has been suggested that bronchoconstriction results in complete closure of air space in the lung [53] and removal of a contractile agonist, such as methacholine from the circulation does not relax the airway smooth muscle fully to its initial level unless physically extended [54]. Furthermore, it has been observed that the functional residual capacity in mice increases in a dose-dependent manner with methacholine usage [55], indicative of gas trapping in the lung and that the structures that are closing are airways rather than alveoli [54].

Parameters G and H, represent energy dissipation, and energy conservation in alveoli, respectively. Parameter G, which is closely related to changes in airway resistance [56, 57], is due to the parenchymal distortion that occurs when the airways constrict. The closure of the airways causes H to increase, due to the reduced lung space in the open regions and this increases the overall elastance [54]. Females showed a significant increase in G and H after infection in all variants except 1A^0^. But the females including 1A^0^ compared to males had opposite results after methacholine challenge showing a significant decrease in both G and H (Figs. 1E, F & 4E, F). These data indicate that for all mouse lines studied there is a clear sex difference in response to various irritants (infection, methacholine), with the exception of 1A^0^ that showed either no sex difference or sex difference depending on the challenge or combination of challenges. We speculate that, the observed no sex difference in G and H parameters for 1A^0^ after infection is due to the incomplete closure of the airways. Whereas, the observed sex differences in Rn for 1A^0^ after infection, were partly due to the narrowing of the airways and perhaps to other unidentified factors. The G can increase due to the heterogeneity in airflow throughout the lung [57, 58] and bronchoconstriction is inherently a heterogeneous phenomenon [59]. Thus a similarity exists between G and Rn after methacholine challenge in SP-A variants. The H can increase due to an increase in intrinsic tissue properties, which could result from the distortion of the parenchyma produced as the airways narrow [56], and increase through the development of regional heterogeneities throughout the lung [56, 57, 60]. The H will be maximal when airway narrowing is maximal and when Rn is maximal, as observed after methacholine challenge; the heterogeneity in the narrowing of airways always seems to develop commensurately with the degree of mean airway constriction [61].

Compliance (Crs) measures the ease with which lung can be inflated, a drop in compliance value indicates increased stiffness in the lungs. The females exhibited a significant decrease in compliance for all groups except the 1A^0^ variant, but following methacholine challenge had a significant increase in Crs in SP-A2 (including 1A^0^variant) (Figs. 1b, & 4b). Data from our previous studies indicated that females are less susceptible to pneumonia and have a better outcome, but are more sensitive to an additional environmental stress (ozone) as shown by a greater reduction in their survival compared to males [45]. Moreover, the bacterial infection caused a significant increase in elastance (Ers) in females except for 1A^0^ variant. Ers measures the elastic rigidity of the lung in the distal airways. The higher respiratory elastance is significantly altered after methacholine challenge with males exhibiting increased elastance including 1A^0^ variant (Figs. 1c, & 4c). The observed lung function differences further confirm that sex differences exist and that the lung microenvironment, as well as, the genetics of innate immunity of an individual, may play an important role in these differences. For example, the 1A^0^ in the absence of methacholine challenge exhibited significantly different values in parameter H, G, Crs, and Ers, from all other mouse lines. Even though we have shown previously that the SP-A variants differ in their bacterial phagocytic activity, with SP-A2 variants exhibiting better phagocytic activity than SP-A1, a higher reduction in the phagocytic activity after ozone exposure compared to non-ozone exposed variants was observed in SP-A2 [36].

Even though, both males and females have similar respiratory requirements, sexual dimorphism in lung function and lung disease has been observed [62]. The sexual dimorphism in the severity of pneumonia [63, 64] and other lung disorders have been observed in different studies [65]. From previous observations and the present lung function data, we postulate that SP-A1 and SP-A2 variants play an important role in the observed sex differences in airway hyperreactivity in response to infection and methacholine challenge and thus sex hormones may differentially affect lung function.

### Genotype differences in response to infection and in response to methacholine

Although, the SP-A1 and SP-A2 variants exhibited major sex difference in most of the lung function studied after infection or in response to methacholine, the SP-A1/SP-A2 (6A^2^/1A^0^, co-ex) showed sex differences with Rrs, Rn, G, H, and Ers being increased and Crs being decreased in females compared to males after infection (Fig. 1), but this pattern changed after methacholine challenge (Fig. 4). Moreover, co-ex exhibited significantly decreased lung function compared to mice with a single gene product i.e. 1A^0^, 1A^3^, 6A^2^, and 6A^4^, in both males and females (Figs. 5, & 6). It has been previously observed that the phagocytic activity of the 1A^0^ variant is higher than the 6A^2^ and the 6A^2^/1A^0^ variants [7]. The structural stability of the SP-A1 (6A^2^) is lower than that of the SP-A2 [25]. Whether the decrease in function in co-ex compared to single gene variants is due to the lower structure stability of 6A^2^ remains to be determined. Moreover, in humans, the ratio of SP-A1 to total SP-A differs significantly in certain diseases such as asthma, cystic fibrosis, and as a function of age [31, 66]. However, some differences have been observed among individuals in the SP-A1/SP-A ratio with no reported lung disease [47], pointing to the possibility that content differences in SP-A1 and SP-A2 may reflect small differences in lung function under non-disease conditions and that these may get magnified in the presence of an insult.

The SP-A2 (1A^0^, 1A^3^) variants displayed a significant difference in airway hyperreactivity compared to SP-A1 (6A^2^, 6A^4^) variants in both males and females with infection and methacholine challenge. Differences between individual SP-A1 and SP-A2 variants were observed. The 1A^0^ male differed from the 6A^2^ male but exhibited similar lung function to 6A^4^ male; the 1A^0^female differed from both 6A^2^ and 6A^4^. Whereas the 1A^3^ males and females exhibited differences with both 6A^2^ and 6A^4^ males and females. The observed gene-specific variant differences in lung functions between SP-A1(6A^2^, 6A^4^) and SP-A2 (1A^0^, 1A^3^) variants may be due to the amino acid differences among the SP-A genes, located within the signal peptide, the collagen-like domain, and the carbohydrate recognition domain (CRD) regions of SP-A. The major amino acid differences that distinguish between SP-A1 and SP-A2 gene-specific variants are located in the collagen-like domain. SP-A1 has a cysteine and SP-A2 has an arginine at position 85 of the precursor molecule. The presence of Cys85 in SP-A1 may create a micro instability when found within a collagen domain [43]. The amino acid differences in the non-collagen domains of SP-A may further contribute to the differences among variants. Whether any of these are responsible for the observed differences in the lung function between SP-A1 and SP-A2 gene-specific variants remains to be determined.

## Conclusion

In summary: 1) the SP-A variants exhibited significant sex differences in lung function mechanics in response to infection alone and to infection plus methacholine; 2) In response to infection alone: a) no significant differences were observed in pressure-volume (PV) curves among the variants in either males or females; b) the SP-A2 (1A^3^) and SP-A1 (6A^2^) exhibited similar inspiratory capacity (*A*) and static compliance (Cst) in co-ex males, but in females, all the variants exhibited similar inspiratory capacity and static compliance; c) the intrinsic elastic properties of the respiratory system (shape constant *k* and area) were similar in both males and females. 3) In response to infection plus methacholine: a) the SP-A2 (1A^3^) gene variant exhibited higher lung function compared to SP-A2 (1A^0^), except for Crs for either sex; b) the SP-A1 (6A^2^, 6A^4^) gene variants exhibited diverse changes in lung function in the parameters studied for both sexes; c) the single gene products exhibited significantly increased lung function mechanics compared to co-ex. Based on our observations, we speculate that if an individual is exposed to inhaled substances, their lung function mechanics will differ depending on the genetics of innate immune molecules such as the SP-A1 and SP-A2 variants.

## Abbreviations

*A*: Estimation of inspiratory capacity
AM: Alveolar macrophage
ANOVA: Analysis of variance
Crs: Compliance
Cst: Static compliance
Ers: Elastance
FOT: Forced oscillation technique
G: Tissue damping
H: Tissue elastance
hTG: Humanized transgenic mice
*k*: Shape constant
KO: Knockout
*Kp*: *Klebsiella pneumoniae*
Rn: Newtonian resistance
Rrs: Resistance
SFTPA1: Gene encoding surfactant protein A1
SFTPA2: Gene encoding surfactant protein A2
SP-A: Surfactant protein A
SP-A1: Surfactant protein A1
SP-A2: Surfactant protein A2
TSB: Tryptic soy broth
